# Photo-Fenton oxidation of cylindrospermopsin at neutral pH with LEDs

**DOI:** 10.1007/s11356-022-23681-7

**Published:** 2022-10-22

**Authors:** David Ortiz, Macarena Munoz, Jorge Garcia, Samuel Cirés, Zahara M. de Pedro, Antonio Quesada, Jose A. Casas

**Affiliations:** 1 Departamento de Ingeniería Química, Universidad Autónoma de Madrid, Ctra. Colmenar km 15, 28049 Madrid, Spain; 2 Departamento de Biología, Universidad Autónoma de Madrid, Ctra. Colmenar km 15, 28049 Madrid, Spain

**Keywords:** Photo-Fenton, Cyanotoxin, Cylindrospermopsin, Degradation, EDDS, LEDs

## Abstract

Cylindrospermopsin (CYN) is a potent cyanobacterial toxin found in freshwaters worldwide. In this work, the feasibility of the photo-Fenton process under neutral pH using light emitting diodes as irradiation source for the removal of this hazardous cyanotoxin from freshwater was investigated. The impact of the kind of iron chelating agent (ethylenediamine-N, N′-disuccinic acid vs. ethylenedinitrilotetraacetic acid) as well as the effect of the main operating conditions viz. H_2_O_2_ dose, Fe(III) load, initial CYN concentration, and Fe(III):EDDS molar ratio on the performance of the process was systematically evaluated. EDDS was selected as the most appropriate iron chelating agent considering the kinetics of the process and the environmental impact (*Vibrio fischeri* and *Artemia salina*). Under optimized conditions ([H_2_O_2_] = 30 mg L^−1^; [Fe(III)] = 5 mg L^−1^; Fe(III):ligand = 1:0.5 (molar ratio)), complete removal of CYN was achieved in 15-min reaction time. Furthermore, the catalytic system showed to be effective in real water matrices (river and reservoir waters) spiked with CYN. Although the presence of inorganic ions (mainly HCO_3_^−^/CO_3_^2−^) and dissolved organic carbon decreased the oxidation rate of CYN due to scavenging reactions and iron coordination, respectively, complete elimination of the cyanotoxin was achieved in all cases. The fate of EDDS along the process was also evaluated to demonstrate that the catalytic system investigated, apart from its effectiveness, warrants the complete absence of residues after reaction. Therefore, the proposed system constitutes a promising method for cyanotoxin treatment either as a drinking water treatment step in conventional plants or as a potential remediation strategy in the natural environment.

**Supplementary information:**

The online version contains supplementary material available at 10.1007/s11356-022-23681-7.

## Introduction

The occurrence of cyanobacterial blooms in surface waters represents an important menace for the environment and public health worldwide. Approximately 50% of these episodes involve the generation of toxic metabolites, called cyanotoxins (Antoniou et al. 2005; Carmichael 1994). Among these hazardous substances, cylindrospermopsin (CYN) has received relatively low attention compared to the well-known microcystins. CYN was originally related to tropical regions, but in the last decade it has been also detected in more temperate climates including subtropical and Mediterranean regions (Al Momani et al. 2008; De la Cruz et al. 2013; Munoz et al. 2021; Preußel et al. 2009; Svirčev et al. 2019). So far, it has been localized in rivers, lakes, and drinking water reservoirs all over the globe: Australia, Israel, Brazil, USA, Asia, and Europe (De la Cruz et al. 2013; Wormer et al. 2008). This cyanotoxin can be produced by different cyanobacterial genera like *Anabaena/Dolichospermum Cylindrospermopsis*, *Umezakia*, *Raphidiopsis*, *Aphanizomenon*, *Chrysosporum*, and *Lyngbya* (Bernard et al. 2016; De la Cruz et al. 2013). Remarkably, CYN is harmful to bacteria, protozoa, plants, invertebrates, and vertebrates including humans, with cytotoxic, hepatotoxic, nephrotoxic, immunotoxic, and genotoxic effects in mammals (De la Cruz et al. 2013). In fact, CYN appears as one of the most toxic cyanotoxins compared to microcystins (MC-LR and MC-RR), anatoxin-*a*, and saxitoxin (Munoz et al. 2019).

While most cyanotoxins are primarily intracellular and the dissolved fraction (extracellular cyanotoxin) is detected in water predominantly once cell lysis occurs, CYN can be released from viable cells along their life cycle (Smith et al. 2008). For instance, whereas extracellular MCs represent less than 10% of total MCs, extracellular CYN has been reported ranging from 40 to 80% of total CYN (Munoz et al. 2021). Furthermore, this cyanotoxin is fairly stable in surface water under sunlight radiation (half-life time of 11 – 15 days) (Chiswell et al. 1999; De la Cruz et al. 2013). Wormer et al. did not observe any sign of biodegradation of CYN exposed to co-ocurring natural bacterial communities from two water bodies during a 40-day study (Wormer et al. 2008). Accordingly, large accumulation of CYN can be expected in water bodies affected by this kind of blooms, posing a significant risk for water uses (consumption and recreation) and for the ecosystem itself.

Drinking Water Treatment Plants (DWTPs) are not specifically designed for the removal of cyanotoxins (Kumar et al. 2018; Pantelić et al. 2013; Park et al. 2018). These plants usually focus on the removal of cyanobacteria without compromising their integrity to prevent cell lysis and, thus, the release of cyanotoxins. Sedimentation, flotation, coagulation/flocculation, sand filtration, and membrane filtration are quite efficient for such goal, allowing to remove 85–99% intact cyanobacterial cells (Munoz et al. 2021). However, as high concentrations of extracellular CYN can be found along the bloom development (usually < 1 – 10 mg L, but concentrations up to 800 mg L^−1^ have been reported) (Humpage and Fastner 2021), the application of advanced treatment processes is required to achieve the elimination of dissolved cyanotoxins as the conventional processes available at the plants, i.e., chlorination and/or adsorption on activated carbon cannot warrant their complete removal (Kumar et al. 2018). In the same line, the search for effective and green processes that allow potential in situ application in the affected water reservoirs would represent an important step forward.

Advanced Oxidation Processes (AOPs) have emerged as promising alternatives for the elimination of cyanotoxins (Munoz et al. 2021). Among them, the Fenton process, based on the catalytic decomposition of hydrogen peroxide using iron salts, is particularly attractive given its environmentally friendly character and relatively low cost. In recent works, the successful application of this process for CYN removal has been demonstrated (Munoz et al. 2019; Schneider et al. 2022). Nevertheless, this technology shows an important shortcoming: the need to operate at acidic conditions (pH_0_ = 3) to avoid iron precipitation, which makes it unattractive for full-scale and/or on-site application. In this context, the use of chelating agents to form active and soluble iron complexes at neutral pH is gaining importance. EDTA (ethylenedinitrilotetraacetic acid) has proved to be widely effective and is by far the mostly used ligand, but it is quite persistent in the environment (De Luca et al. 2014; Zhang et al. 2008). In the last years, EDDS (ethylenediamine-N, N′-disuccinic acid) has been proposed as an environmentally benign replacement for EDTA given its ready biodegradability (Tandy et al. 2006; Vandevivere et al. 2001). All in all, the complexation of iron by chelating agents and the operation at neutral pH unavoidably reduce the rate of the process. Accordingly, the intensification of the system is required. The application of irradiation (photo-Fenton) is particularly attractive as it allows to overcome the slow redox cycle of iron and, thus, enhance the production of HO_x_^·^. In particular, the use of EDDS as a chelating agent leads to the formation of a highly stable dissolved complex with Fe(III) in a wide pH range of 3–9 (Huang et al. 2012). However, in the presence of light, this complex can be broken, releasing ligand-free Fe(II) and the EDDS^•^ radical into the medium, which can favor the generation of new highly oxidizing radicals (Ahile et al. 2020; Miralles-Cuevas et al. 2019; López-Vinent et al; 2022). The main shortcoming is related to the electrical consumption, but it can be substantially reduced using light emitting diodes (LEDs). Furthermore, they show other benefits like long lifetime, no overheating, and absence of mercury content.

The application of photo-Fenton promoted with Fe(III)–EDDS has proved to be quite effective for the removal of different emerging pollutants (Gonçalves et al. 2020; López-Vinent et al. 2020a, b; Miralles-Cuevas et al. 2019). Nevertheless, to the best of our knowledge, it has not been applied so far for the elimination of cyanotoxins. In this work, the feasibility of the photo-Fenton process assisted with LED irradiation at neutral pH and ambient conditions for the degradation of CYN has been investigated. Both EDTA and its environmentally benign isomer EDDS chelating agents were tested to complex iron and prevent its precipitation as iron hydroxides along reaction. Their potential impact on the environment was assessed by analyzing their toxicity using target organisms of different trophic levels (namely the bacterium *Vibrio fischeri* and the invertebrate *Artemia salina*). The fate of EDDS along the oxidation process was also investigated to confirm its breakdown and the absence of residues after the reaction. Once the most appropriate chelating agent was selected, a complete operating conditions study was carried out to analyze the influence of initial H_2_O_2_ dose, Fe(III) load, initial CYN concentration, and Fe(III):EDDS molar ratio on the kinetics of CYN oxidation. As a proof of concept, the performance of the catalytic system was tested in different real waters (reservoir and river waters).

## Materials and methods

### Materials and chemicals

CYN (MW: 415.4 g mol^−1^, ≥ 99%) was provided by Laboratorio CIFGA S.A. (Spain). The main properties of the cyanotoxin together with the oxidation stoichiometry are provided in Table S1 of the Supplementary Material. EDDS (35%) and EDTA (97%) were purchased from Sigma-Aldrich. Hydrogen peroxide solution (30% w/w) and iron (III) nitrate nonahydrate (98%) were supplied by Panreac. All these compounds were used as received without further purification. Deionized water was used to perform the experiments.

### Typical reaction procedure

Photo-Fenton oxidation runs were carried out at ambient conditions (25 °C, 1 atm) and natural pH (pH_0_ = 6.7 – 7.2) in 20-mL glass batch reactors, equipped with a stirrer (750 rpm) and temperature control. The external wall and the top of the reactor were surrounded by a commercial LED strip (SMD2835 180 LEDs m^−1^). The horizontal and vertical irradiance values were 60 and 87 W m^−2^, respectively. LED light emitted at 6400 K with a power of 8 W m^−1^ (*λ* = 380–780 nm). The impact of the main operating conditions viz. type of iron chelating agent (EDDS vs. EDTA), H_2_O_2_ dose (0.4 – 50 mg L^−1^), iron concentration (1 – 7 mg L^−1^), initial CYN concentration (10 – 200 mg L^−1^), and Fe(III):EDDS molar ratio (1:0.25 – 1:3) was systematically evaluated. To distinguish the contribution of light, H_2_O_2_, and Fe(III)–EDDS to the reaction, blank experiments were performed. All runs were performed in triplicate being the standard deviation less than 10% in all cases.

### Analytical methods

CYN concentration was determined by a high-performance liquid chromatograph equipped with a diode array detector (Shimadzu, mod. Prominence-i, LC-2030C LT; SPDM30A). An Eclipse Plus C18 column (Agilent) was used as stationary phase and deionized water as mobile phase at a flow rate of 0.8 mL min^−1^. Analyses were carried out at 262 nm. A mixture of 50/50% (v/v) of hydrochloric acid aqueous solution (1 mM, pH 3.0) and ammonium sulfate aqueous solution (5 mM, pH 2.65) was used for the analyses of EDDS at a flow rate of 1.0 mL min^−1^. Quantification was carried out at 207 nm. H_2_O_2_ and dissolved iron concentrations were measured by colorimetry using a UV 2100 Shimadzu UV–VIS spectrophotometer following the methods of titanium sulfate (Eisenberg 1943) and *o*-phenantroline (Sandell 1959), respectively. Total organic carbon (TOC) and inorganic carbon (IC) were measured using a TOC analyzer (Shimadzu, mod. TOC VSCH).

### Toxicity study

The toxicity of the iron ligands tested in this work (EDDS and EDTA) was evaluated using the bacterium *Vibrio fischeri* and the invertebrate *Artemia salina* as target organisms. The procedures followed for both kinds of tests are based on previous works (Munoz et al. 2019, 2011; Vanhaecke et al. 1981).

The determination of the toxicity of the ligands using *Vibrio fischeri* was performed by the Microtox test (ISO 11348–3, 1998). The pH (6 – 8) and salinity (2% NaCl solution) of the samples were previously adjusted, and their bioluminescence was measured after 15 min of exposure at 15 °C using a photomultiplier M500 Microtox Analyzer (Azur Environmental). The effective concentration (EC_50_) of EDDS and EDTA, defined as the concentration of the compound that reduces the light emission intensity by 50%, was determined.

*Artemia salina* cysts (1 g) were incubated under continuous illumination and aeration (0.5 L synthetic seawater at 30 °C). Cysts hatched within 24 h under these conditions. Acute toxicity was then determined under the following standard conditions: salinity (35 g L^−1^), pH = 7, 30 °C, and darkness. The vials containing 20 nauplii in 2 mL of sample were incubated for a period of 72 h, using three replicates of each test concentration. Nauplii were considered dead if after 10 s of observation no movement was observed. The lethal concentration (LC_50_), defined as the lethal concentration of the compound that kills 50% of the nauplii within 72 h, was used to determine the toxicity of the ligands.

## Results and discussion

### The choice of chelating agent

EDDS and EDTA were tested as Fe(III) chelating agents. Iron complexes were prepared by the addition of the corresponding ligand to a solution of dissolved ferric iron at a Fe(III):chelating molar ratio agent of 1:2 at ambient temperature without pH adjustment. Unless otherwise indicated, the concentration of iron was 5 mg L^−1^. The mixture was stirred for 5 min prior being used in reaction. The UV/Vis spectra of the complexes, which were consistent with the previously reported in the literature (Huang et al. 2012; Kocot et al. 2006; Li et al. 2010), are collected in the Fig. S1 of the Supplementary Material. The Fe(III)–EDTA and Fe(III)–EDDS chelated complexes showed an absorption limit slightly higher to 400 nm. However, a slight absorbance up to 450 nm has also been observed for both compounds. These results are in good agreement with those reported by López-Vinent et al. (2022), who observed absorbance edges close to 450 and 500 nm for the Fe(III)–EDTA and Fe(III)–EDDS complexes, respectively.

**Fig. 1:**
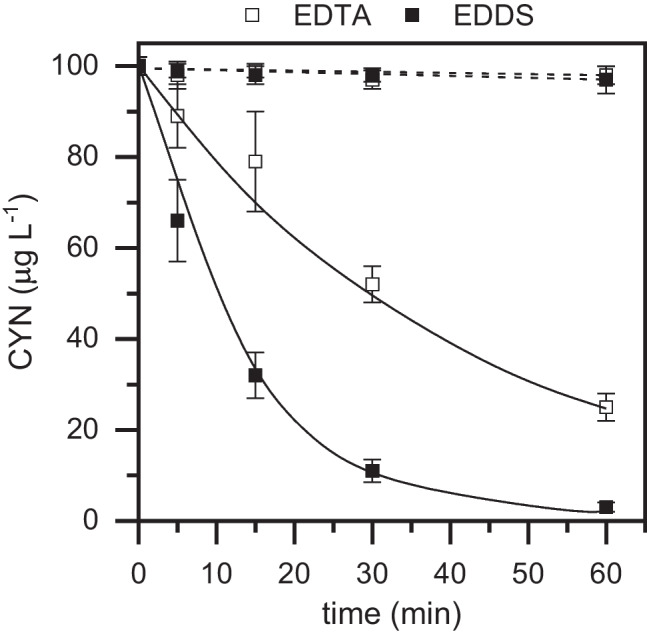
Evolution of CYN upon dark and photo-Fenton oxidation with LED using EDTA and EDDS as ligands ([CYN]_0_ = 100 µg L^−1^; [H_2_O_2_] = 30 mg L^−1^; [Fe(III)] = 5 mg L^−^.^1^; Fe(III):ligand = 1:2 (molar ratio); pH_0_ ~ 7; *T* = 25 °C). Experimental (symbols) and model fit (solid and dash lines for photo-assisted and dark experiments, respectively)

The results obtained in the dark and photo-Fenton oxidation of CYN (100 mg L^−1^) using Fe(III)–EDDS and Fe(III)–EDTA complexes are collected in Fig. 1. As can be observed, light irradiation appears to be a crucial factor in the catalytic process. Reactions in the absence of light showed a degradation yield lower than 5% after 1-h reaction time with both chelating agents. Furthermore, evaluating the photo-assisted experiments, Fe(III)–EDDS was a clearly more effective photo-catalyst than Fe(III)–EDTA under the operating conditions tested. While almost complete degradation (> 99%) of CYN was achieved with Fe(III)–EDDS after 1-h reaction time, only 75% conversion was reached using Fe(III)–EDTA.

The contribution of light can be explained by the reactions governing the process based on several works (López-Vinent et al. 2022; Miralles-Cuevas et al. 2019). While in the absence of light the Fe(III)–ligand complex is highly stable, in the presence of light these complexes exhibit high catalytic activity. Hydrogen peroxide is adsorbed on the iron complex and decomposed in a redox cycle (r1–4). This is a slow process but catalyzed by light. Additionally, the iron complex can be broken by the action of light (r5), releasing ligand-free Fe(II) and the metal-free ligand radical into the medium. Finally, metal-free ligand radicals can interact with H_2_O_2_ present in the medium, favoring the production of additional hydroxyl radicals (r6).

r1 $$\mathrm{L}-\mathrm{Fe}\left(\mathrm{III}\right)+{\mathrm{H}}_{2}{\mathrm{O}}_{2}\to \mathrm{L}-\mathrm{Fe}\left(\mathrm{III}\right)-{\mathrm{H}}_{2}{\mathrm{O}}_{2}$$

r2 $$\mathrm L-\mathrm{Fe}\left(\mathrm{III}\right)-{\mathrm H}_2{\mathrm O}_2\xrightarrow{\mathrm{hv}}\mathrm L-\mathrm{Fe}\left(\mathrm{II}\right)+\mathrm{HOO}^\bullet+\mathrm H^+$$

r3 $$\mathrm{L}-\mathrm{Fe}\left(\mathrm{II}\right)+{\mathrm{H}}_{2}{\mathrm{O}}_{2}\to \mathrm{L}-\mathrm{Fe}\left(\mathrm{II}\right)-{\mathrm{H}}_{2}{\mathrm{O}}_{2}$$

r4 $$\mathrm L-\mathrm{Fe}\left(\mathrm{II}\right)-{\mathrm H}_2{\mathrm O}_2\xrightarrow{\mathrm{hv}}\mathrm L-\mathrm{Fe}\left(\mathrm{III}\right)+\mathrm{HO}^\bullet+\mathrm{OH}^-$$

r5 $$\mathrm{Fe}\left(\mathrm{III}\right)-\mathrm L\xrightarrow{\mathrm{hv}}\mathrm{Fe}\left(\mathrm{II}\right)+\mathrm L^\bullet$$

r6 $$\mathrm L^\bullet+{\mathrm H}_2{\mathrm O}_2\rightarrow\mathrm L-\mathrm{OH}+\mathrm{HO}^\bullet$$

This mechanism would explain the evolution of the CYN oxidation process both in the presence and absence of light. The experimental data of the photo-assisted experiments were successfully described by a pseudo-first order kinetic equation, obtaining apparent rate constant values of 0.076 and 0.024 min^−1^ for Fe(III)–EDDS and Fe(III)–EDTA, respectively, while this value was close to 0 min^−1^ in dark condition.

The obtained results are consistent with those recently reported by López-Vinent et al. in the photo-Fenton oxidation of propranolol with LEDs by EDDS and EDTA iron complexes (López-Vinent et al. 2020a, b). They are also in good agreement with the findings of Huang et al., who observed a higher degradation rate of 2,2-bis-(4-hydroxyphenyl)propane (BPA) in the presence of Fe(III)–EDDS compared to Fe(III)–EDTA and other complexes such as Fe(III)–oxalate and Fe(III)–citrate (Huang et al. 2012). Although the effectiveness of the iron complexes seems to greatly depend on the nature of the target pollutant to be treated (López-Vinent et al. 2020a, b), another reason behind the better performance of Fe(III)–EDDS compared to Fe(III)–EDTA may be related to their redox potentials. Huang et al. (2012) determined the redox potentials of both complexes by cyclic voltammetry and demonstrated that the half-wave potential of Fe(III)–EDTA was slightly higher than that of Fe(III)–EDDS (0.098 and 0.069 V/NHE, respectively). Apart from this reason, it must be noted that the Fe(III)–EDDS species showed also a higher activity for H_2_O_2_ decomposition (45% and 30% H_2_O_2_ decomposition yields were obtained during the CYN oxidation with Fe(III)–EDDS and Fe(III)–EDTA, respectively) and, thus, for the generation of hydroxyl radicals to oxidize CYN.

Apart from considering the activity of the iron complexes, their environmental impact was also evaluated. For such goal, the toxicity of both EDDS and EDTA was analyzed using living organisms of different trophic levels (the bacterium *Vibrio fischeri* and the microcrustacean *Artemia salina*). In both cases, EDDS was significantly less toxic than EDTA. EC_50_ (*Vibrio fischeri*) and LC_50_ (*Artemia salina*) values of 495 and 52 mg L^−1^ were obtained for EDDS, respectively, whereas EDTA showed considerably lower values viz. 75 and 1.1 mg L^−1^, respectively. These results allow to confirm that the impact of the widely used chelating agent EDTA in the environment is considerably stronger than that of its isomer EDDS. This is consistent with previous literature in the field, where EDDS was identified as a safe and environmentally benign alternative for EDTA (Li et al. 2010; López-Vinent et al. 2020a, b; Miralles-Cuevas et al. 2019; Zhang et al. 2008). Considering the better catalytic performance of Fe(III)–EDDS and also its significantly lower toxicity compared to Fe(III)–EDTA, it was selected to proceed with the study.

Although the concentration of EDDS used is remarkably lower than the LC_50_ value, its removal would be desirable. Remarkably, it was found that EDDS is also degraded along photo-Fenton oxidation at a rate around three times slower than that observed for the target pollutant. These results are desirable as the iron complex is available while it is required for CYN elimination, but it is afterwards removed. Consequently, it can be confirmed that no EDDS residues would remain in water after the application of the process (see experimental data in Fig. S2 of the Supplementary Material). This is also an important advantage of EDDS compared with EDTA. For instance, Metsärinne et al. (2001) investigated the photodegradation of EDTA and EDDS within natural UV radiation range in lake water and demonstrated that the photodegradation of EDDS is markedly faster than that of EDTA in both laboratory and field experiments.

### Operating conditions study

**Fig. 2:**
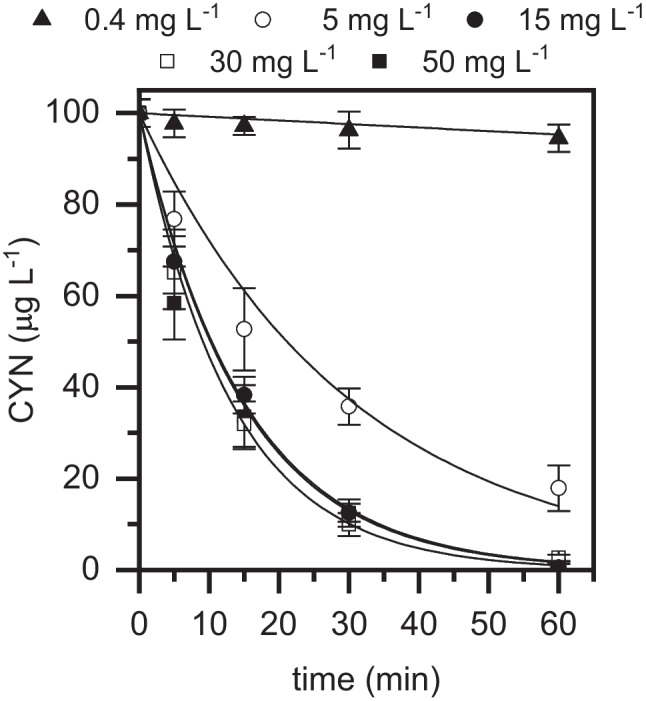
Evolution of CYN upon photo-Fenton oxidation with LED at different H_2_O_2_ doses ([CYN]_0_ = 100 µg L^−1^; [Fe(III)] = 5 mg L^−^.^1^; Fe(III):EDDS = 1:2 (molar ratio); pH_0_ ~ 7; *T* = 25 °C). Experimental (symbols) and model fit (solid lines)

The impact of the main variables of the process, i.e., H_2_O_2_ dose, iron concentration, initial CYN concentration and Fe(III):chelating agent molar ratio were systematically evaluated. The influence of the H_2_O_2_ dose on the photo-Fenton oxidation of CYN can be seen in Fig. 2. The stoichiometric dose of H_2_O_2_ (0.4 mg L^−1^) led to a negligible conversion of CYN after 1 h reaction time. Clearly, the increase of H_2_O_2_ concentration enhanced the degradation of CYN. A dose of 5 mg L^−1^ allowed to reach 80% removal of the cyanotoxin, while doses of 15, 30, and 50 mg L^−1^ warranted its complete elimination. In fact, H_2_O_2_ is able to increase the regeneration of Fe(III) from Fe(II) and, thus, increase the generation of hydroxyl radicals. Above a threshold value of 30 mg L^−1^, the oxidation rate of CYN showed a slight decrease (0.076 and 0.067 min^−1^ apparent rate constant values were obtained with 30 and 50 mg L^−1^, respectively). This is due to the fact that, at high excess of H_2_O_2_, the oxidant can act as HO· scavenger, reacting with this radical and giving rise to an oxidation reaction with less active radical as product (HOO·). Both radicals (HO· and HOO·) can finally lead to termination reactions producing molecular oxygen (Pera-Titus et al. 2004). Based on these results, a H_2_O_2_ dose of 30 mg L^−1^ was selected for further experiments although 15 mg L^−1^ would be also a suitable amount to perform the process.

**Fig. 3:**
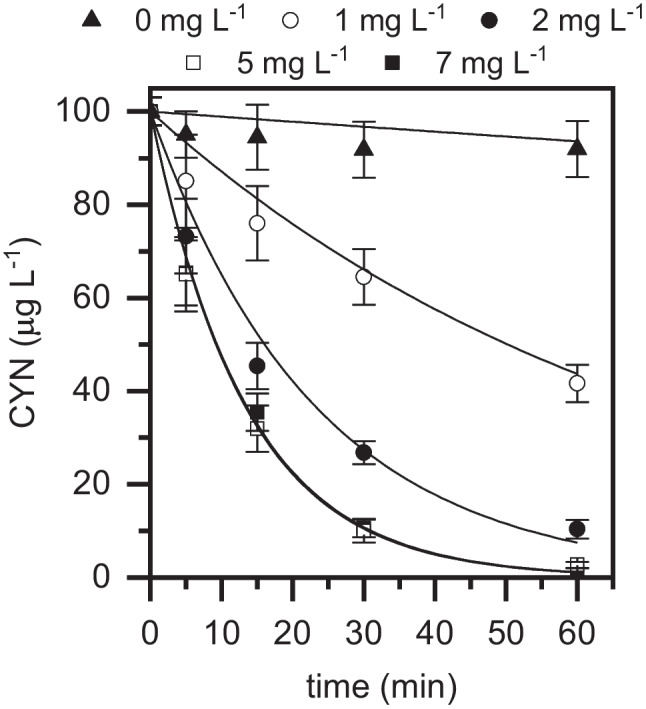
Evolution of CYN upon photo-Fenton oxidation with LED at different Fe(III) concentrations ([CYN]_0_ = 100 µg L^−1^; [H_2_O_2_] = 30 mg L^−^.^1^; Fe(III):EDDS = 1:2 (molar ratio); pH_0_ ~ 7; *T* = 25 °C). Experimental (symbols) and model fit (solid lines)

The impact of iron concentration on the degradation of CYN was investigated in the range of 1 – 7 mg L^−1^. As can be seen in Fig. 3, in the absence of catalyst, almost negligible disappearance of CYN was observed, which allowed to confirm that the contribution of H_2_O_2_ or H_2_O_2_ photolysis to CYN oxidation can be neglected under these operating conditions. Clearly, the oxidation rate of CYN significantly increased with catalyst dosage up to 5 mg L^−1^. A further increase of catalyst up to 7 mg L^−1^ slightly decreased the oxidation rate due to scavenging reactions where an excessive hydroxyl and hydroperoxyl concentration generated in the Fe redox cycle leads to termination reactions (Pera-Titus et al. 2004). It is then crucial to reach a compromise considering the concentrations of catalyst, H_2_O_2_, and pollutant initial concentration. Based on the obtained results, 5 mg L^−1^ was selected as catalyst concentration in this study.

Remarkable fluctuations in cyanotoxin concentration are expected in the water reservoirs affected by cyanobacterial blooms as it is a predominantly seasonal phenomenon, mainly influenced by nutrient availability and water temperature (Whitton and Potts 2012). Furthermore, the bloom is a sequential process that involves growth, maintenance, and decay stages (Li et al. 2020; Tang et al. 2018). For these reasons, analyzing the influence of the initial concentration of cyanotoxin in the photo-Fenton process is essential. For such goal, different CYN concentrations in the range of 10 – 200 mg L^−1^ were tested. The results obtained are depicted in Fig. S3 of the Supplementary Material. Complete degradation of the cyanotoxin was achieved along the 1-h experiment regardless of the initial concentration of CYN, which demonstrates the feasibility of this process for cyanotoxin removal from water throughout the bloom event. These results are consistent with the fact that the experimental data were successfully described by a pseudo-first order kinetic equation, obtaining essentially the same apparent rate constant value in the CYN concentration range evaluated (approx. 0.076 min^−1^).

**Fig. 4:**
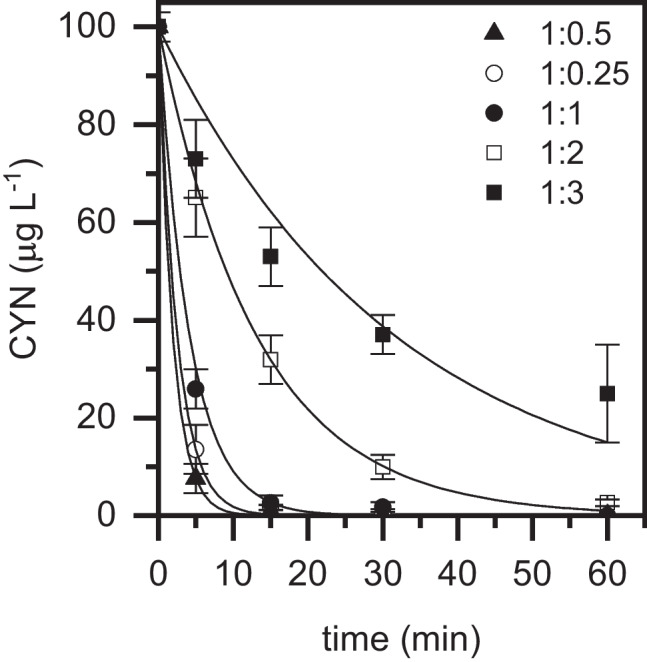
Evolution of CYN upon photo-Fenton oxidation with LED at different Fe(III):EDDS molar ratios ([CYN]_0_ = 100 µg L^−1^; [H_2_O_2_] = 30 mg L^−1^; [Fe(III)] = 5 mg L^−^.^1^; pH_0_ ~ 7; *T* = 25 °C). Experimental (symbols) and model fit (solid lines)

A crucial aspect to consider in the photo-Fenton process catalyzed by iron complexes is the iron:chelating agent molar ratio. Although EDDS plays a key role in the catalytic system as it enhances the solubility and stability of iron in aqueous solution at neutral pH, any reduction in its consumption is desirable from both environmental and economic points of view. A standard Fe(III):EDDS molar ratio of 1:2 was used in this work taking into account previous studies where it was demonstrated that the excess of chelating agent above the stoichiometric ratio (1:1) (Li et al. 2010) improves the rate of the process (De Luca et al. 2014; Gonçalves et al. 2020). Under these conditions, free chelators could recapture the iron ions released from the destroyed complex, avoiding iron precipitation (De Luca et al. 2014). All in all, to evaluate the effect of Fe(III):EDDS molar ratio under the operating conditions tested in this work, it was varied from 1:0.25 to 1:3. As can be seen in Fig. 4, the decrease in the Fe(III):EDDS molar ratio up to 1:0.5 progressively increased the kinetics of the process. This somehow unexpected result can be explained by the competition for HO· between the target pollutant and the EDDS excess. It must be noted that, in the current work, the difference in concentration between the target pollutant and EDDS is particularly significant considering that CYN initial concentration was only 100 mg L^−1^ and that of chelating agent was in the range of 6.4 to 51.3 mg L^−1^ (chelating agent:target pollutant molar ratios of 22:0.24 and 176:0.24 mM, respectively). This contrasts with previous works, where typical chelating agent:target pollutant molar ratios were much closer (Gonçalves et al. 2020; López-Vinent et al. 2020a, b). For instance, López-Vinent et al. used equimolar amounts of EDDS and target pollutant in the oxidation of propranolol (López-Vinent et al. 2020a, b). In those works, a minimum Fe(III):EDDS molar ratio of 1:1 was selected as optimum. On the contrary, our results are in good agreement with the reported by Cui et al. (2017), who also found that a Fe(III):EDDS molar ratio of 1:0.5 was the optimum for the photo-Fenton oxidation of ethylbenzene. Consistent with our findings, these authors demonstrated that the chelating agent:target pollutant molar ratio shows a great influence on the oxidation performance. In particular, they observed that the kinetics of the process was progressively enhanced by increasing this molar ratio from 1:1 to 1:4. Therefore, it can be confirmed that the improvement in Fe(III) chelation promoted by EDDS can be counterbalanced if large amounts of ligand are present in solution. Furthermore, Cui et al. (2017) also hypothesized that the excessive protection of Fe(III) by EDDS could also inhibit the availability of iron.

**Fig. 5:**
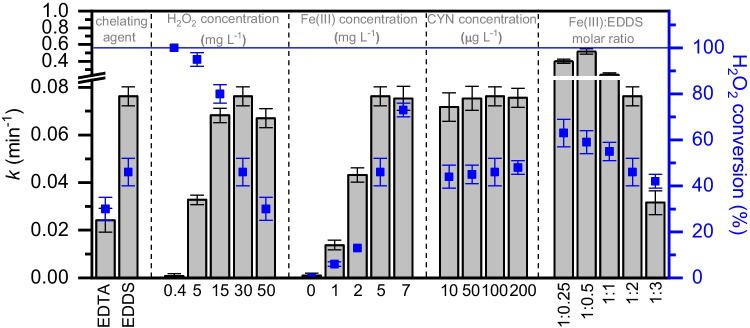
Apparent pseudo-first order kinetic constant and H_2_O_2_ conversion values obtained in the photo-Fenton oxidation of CYN with LED at different operating conditions (standard conditions: [CYN]_0_ = 100 mg L^−1^; [H_2_O_2_] = 30 mg L^−1^; [Fe(III)] = 5 mg L^−^.^1^; Fe(III):EDDS = 1:2 (molar ratio); pH_0_ ~ 7; *T* = 25 °C)

As has been mentioned along the discussion of the results obtained in the operating conditions study, the experimental data were successfully described by a pseudo-first order kinetic equation. As a summary, Fig. 5 collects the apparent rate constant values obtained at the different operating conditions tested. The H_2_O_2_ conversion values achieved at the end of each reaction are also collected. Briefly, the choice of chelating agent showed a significant impact on the degradation rate of CYN, which was up to three times faster with Fe(III)–EDDS compared to Fe(III)–EDTA. Consistent with these results, the H_2_O_2_ conversion values achieved were 45% and 30%, respectively. H_2_O_2_ and Fe(III) concentrations also had a great impact on the rate of the process. The apparent rate constant values progressively increased with the increase of H_2_O_2_ and Fe(III) concentrations up to of 30 and 5 mg L^−1^, respectively. Above these threshold values, the apparent rate constants were slightly decreased due to scavenging reactions. Regarding H_2_O_2_ conversion, it progressively decreased with the increase of H_2_O_2_. Doses in the range of 15 – 30 mg L^−1^ warranted the fast removal of CYN, while doses below limited the rate of the process and doses above led to an inefficient consumption of the reagent. On the other hand, the increase of catalyst led to a progressive increase of H_2_O_2_ conversion. Remarkably, and consistent with the pseudo-first order kinetics, the initial concentration of CYN did not affect the oxidation rate constant. In the same line, H_2_O_2_ conversion values were practically the same regardless of the initial cyanotoxin concentration. Finally, the Fe(III):EDDS molar ratio also showed a substantial influence on the oxidation rate of CYN, being a molar ratio of 1:0.5 the optimum one as it warranted the chelation of iron and also prevented the competitive consumption of H_2_O_2_ by EDDS, which was observed with molar ratios at or above 1:1.

To further optimize the operating conditions of the process, a new set of experiments was carried out analyzing the effect of H_2_O_2_ and Fe(III) at a Fe(III):EDDS molar ratio of 1:0.5. Response surface methodology, in particular central composite rotational design (CCRD), was employed to determine the optimum doses of these reagents (see Supplementary Material for all experimental data). Based on this model, the dependence of the apparent pseudo-first order kinetic constant value with the operating conditions (H_2_O_2_ and Fe(III) concentration) was described according to the following polynomial equation:

$$k=\left({min}^{-1}\right)=-3.38\cdot10^{-2}+4.23\cdot10^{-2}\cdot A-0.39\cdot10^{-2}\cdot B+0.16\cdot10^{-2}\cdot A\cdot B$$

where A is the concentration of Fe(III) catalyst (mg L^−1^), and B is the H_2_O_2_ dose (mg L^−1^).

According to the obtained results, the best performance of the system was achieved at the highest doses of catalyst and oxidant (7 and 50 mg L^−1^, respectively). However, attending to both, economic criteria as well as the applicability of the process as a remediation treatment, the operating conditions for further studies were selected as those that ensured the complete degradation of CYN in less than 15 min employing the lowest doses of catalyst and oxidant (5 and 30 mg L^−1^, respectively).

### Proof of concept: application to real water matrices

**Fig. 6:**
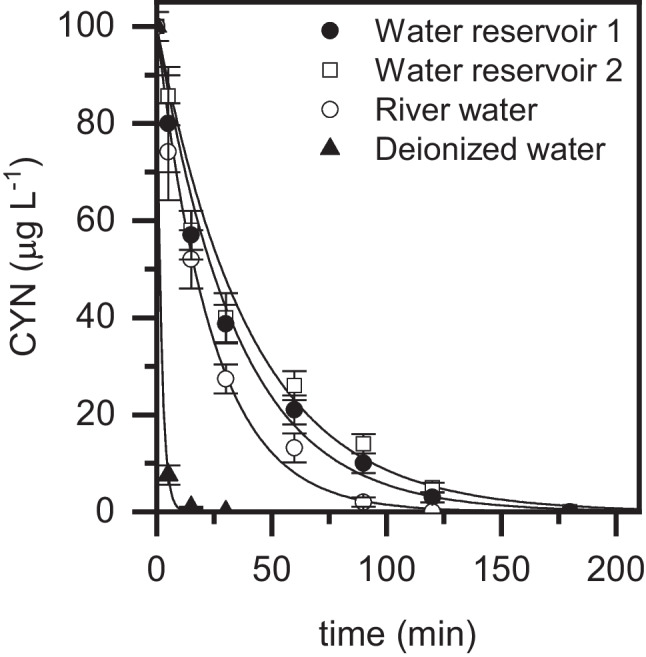
Evolution of CYN upon photo-Fenton oxidation with LED in different real water matrices ([CYN]_0_ = 100 µg L^−1^; [H_2_O_2_] = 30 mg L^−1^; [Fe(III)] = 5 mg L^−^.^1^; Fe(III):EDDS = 1:0.5 (molar ratio); pH_0_ ~ 7; *T* = 25 °C). Experimental (symbols) and model fit (solid lines)

To further demonstrate the feasibility of the photo-Fenton process with LED catalyzed by the Fe(III)–EDDS complex for the removal of CYN, it was applied to real water matrices (two water reservoirs and a river) spiked with the cyanotoxin. All these samples were previously filtered (0.45 mm PTFE). The representative characterization of the matrices is collected in Table 1. Based on the results obtained in the operating condition study, oxidation runs were performed with a H_2_O_2_ concentration of 30 mg L^−1^, a Fe(III) dose of 5 mg L^−1^, and a Fe(III):EDDS molar ratio of 1:0.5. The initial concentration of CYN was established at 100 mg L^−1^. The obtained results are provided in Fig. 6 (see Fig S6 for the apparent pseudo-first order kinetic constant and H_2_O_2_ conversion). Clearly, the catalytic system was effective for the cyanotoxin removal regardless of the composition of the water matrix although it clearly affected its oxidation rate. In general, the oxidation proceeded considerably slower in the real water matrices compared to deionized water. Apparent pseudo-first order rate constant values of 0.516, 0.042, 0.0288, and 0.0245 min^−1^ were obtained for deionized, river, reservoir 1, and reservoir 2 waters, respectively. On the other hand, H_2_O_2_ conversion was also significantly lower in the real matrices (59%, 35%, 11%, and 8% were achieved in deionized, river, and reservoir 1 and 2 waters, respectively).

**Table 1:** Representative analysis of the real water matrices tested

Parameter	Reservoir 1	Reservoir 2	River
pH	7.2	6.9	7.1
TOC (mg L^−1^)	6.2	6.1	2.7
IC (mg L^−1^)	26.3	25.8	12.8
Conductivity (mS cm^−1^)	72	160	180
Cl^−^ (mg L^−1^)	1.5	1.8	14.1
SO_4_^2−^ (mg L^−1^)	3.1	6.7	11.2

The obtained results can be explained by the occurrence of scavenging reactions where hydroxyl radicals are consumed by co-existing substances in the water matrix, but also by the likely interaction of iron with dissolved organic matter. The presence of relevant concentrations of HCO_3_^−^/CO_3_^2−^ (measured as inorganic carbon) in the real water matrices seems to be an important reason behind the activity decline as these species are well-known hydroxyl radical scavengers (Lumbaque et al. 2019). In fact, it allows to explain the differences found among the real waters treated. Clearly, the oxidation was much faster in the river sample compared to the ones from the reservoirs. In fact, its inorganic carbon concentration corresponded to approximately half of the measured in the reservoirs. On the other hand, it must be also noted that the TOC concentration was also two times higher in the reservoir water compared to the river one. Humic acids are usually identified as the major component of dissolved organic matter in natural waters (He et al. 2010; Park et al. 2017). These complex substances usually contain carboxylated, phenolic, and carbonyl functional groups, which can interact with iron, leading to reduced Fenton oxidation efficiency. This finding is consistent with the significantly lower H_2_O_2_ conversion achieved in the real waters and can also justify the differences among them. Finally, the slight variations found between the samples from reservoirs 1 and 2 could be related to the presence of other relevant inorganic ions. The conductivity value, in particular the concentrations of Cl^−^ and SO_4_^2−^, was somehow higher in reservoir 2. These species are also well-known hydroxyl radical scavengers and, although they can lead to the generation of chlorine and sulfate radicals, respectively, they are significantly less effective than hydroxyl radicals (Lumbaque et al. 2019). Furthermore, Cl^−^ and SO_4_^2−^ could also interact with the Fe(III) complex (Lin et al. 2010; Miralles-Cuevas et al. 2014) leading to the significantly less effective complexes for H_2_O_2_ decomposition.

Finally, although the process has shown a high efficiency in the degradation of cyanotoxins under LED light, a new experiment was carried out using sunlight as irradiation source (650 W m^−2^) in order to evaluate a more realistic scenario for the degradation of CYN in a natural environment (Figure S5 of the Supplementary Material). In this experiment, the complete degradation of the cyanotoxin was achieved in less than 5 min. Comparing this result with that obtained using LED light at the same time (80% degradation of CYN), the potential of the process as a remediation system for cyanotoxin removal in natural environments is evident.

## Conclusions

The photo-Fenton process promoted by Fe(III)–EDDS complexes under neutral pH using LEDs as irradiation source has proved to be effective for the removal of CYN from water. EDDS constitutes a promising alternative for the conventional EDTA chelating agent, showing a higher activity in the process together with a more environmentally friendly character. Furthermore, EDDS is completely removed after the oxidation reaction, warranting the absence of residues in water after the application of the process. The catalytic system is clearly influenced by the H_2_O_2_ dose, iron concentration, and Fe(III):EDDS molar ratio, while the initial concentration of CYN does not have any impact on the oxidation rate. Remarkably, the system showed a reasonable activity in real water samples from a river and two water reservoirs. Nevertheless, the occurrence of inorganic ions (mainly HCO_3_^−^/CO_3_^2−^) and dissolved organic carbon led to a decrease on the oxidation rate of CYN due to radical scavenging reactions and iron coordination, respectively. All in all, the developed catalytic system represents a promising technology for the removal of CYN from water, which opens the door for its potential application either as a drinking water treatment step in conventional plants or even as a remediation strategy in the natural environment after careful evaluation of its environmental impact.

## Supplementary information

Below is the link to the electronic supplementary material.

Supplementary file1 (DOCX 686 KB)

## Data Availability

All data and materials have been provided within the manuscript.
