# Supplementary material for: Photo-Fenton oxidation of cylindrospermopsin at neutral pH with LEDs
Source: Environ Sci Pollut Res Int. 2022 Oct 22;30(8):21598–607. doi: 10.1007/s11356-022-23681-7 (PMC9938050; doi:10.1007/s11356-022-23681-7)
Supplement: Supplementary file 1 — Supplementary file1 (DOCX 686 KB) [file 11356_2022_23681_MOESM1_ESM.docx]

**Supplementary Material**

**Photo-Fenton oxidation of cylindrospermopsin at neutral pH with LEDs**

David Ortiz^1,^*, Macarena Munoz^1,^*, Jorge Garcia^1^, Samuel Cirés^2^, Zahara M. de Pedro^1^, Antonio Quesada^2^ and Jose A. Casas^1^

^1^Departamento de Ingeniería Química, Universidad Autónoma de Madrid, Ctra. Colmenar km 15, 28049 Madrid, Spain

^1^Departamento de Biología, Universidad Autónoma de Madrid, Ctra. Colmenar km 15, 28049 Madrid, Spain

*Corresponding author phone: +34 91 497 3765; e-mail: [david.ortiz@uam.es](mailto:david.ortiz@uam.es); [macarena.munnoz@uam.es](mailto:macarena.munnoz@uam.es)

**Table S1.** Main properties of CYN and theoretical stoichiometry for its oxidation.

|  | **CYN** |
| --- | --- |
| **Molecular structure** |  |
| **Molecular weight (g mol^-1^)** | 415.4 |
| **Reaction** | C_15_H_21_N_5_O_7_S + 49 H_2_O_2_ 🡪 15 CO_2_ + 56 H_2_O + 5 HNO_3_ + H_2_SO_4_ |
| **H_2_O_2_ (mg L^-1^)*** | 0.4 |

^*^ Theoretical stoichiometric dose of H_2_O_2_ for the complete oxidation of CYN (initial concentration = 100 μg L^-1^).

**Fig S1**. UV-visible absorption spectra of Fe(III)-EDDS and Fe(III)-EDTA complexes.

**Fig. S2**. Evolution of CYN upon photo-Fenton oxidation with LED in different real water matrices ([CYN]_0_ = 100 μg L^-1^; [H_2_O_2_] = 30 mg L^-1^; [Fe(III)] = 5 mg L^-1^; Fe(III):EDDS = 1:1 (molar ratio); pH_0_ ~ 7; T = 25 ºC). Experimental (symbols) and model fit (solid lines).

**Fig. S3.** Impact of the initial concentration of CYN on its degradation upon photo-Fenton oxidation with LED ([H_2_O_2_] = 30 mg L^-1^; [Fe(III)] = 5 mg L^-1^; Fe(III):EDDS = 1:2 (molar ratio); pH_0_ ~ 7; T = 25 ºC). Experimental (symbols) and model fit (solid lines).

**Optimization of catalyst and oxidant doses**

A statistical analysis based on response surface technology (RSM) coupled with central composite rotational design (CCRD) was applied to evaluate and optimize the doses of H_2_O_2_ and Fe(III) at a Fe(III):EDDS molar ratio of 1:0.5. Table S2 shows the levels of the variables (low, center and high), denoted as -1, 0 and 1, respectively. Table S3 shows the apparent pseudo-first order kinetic constants obtained while the response surface is depicted on Figure S4. The fit between the model and the experimental data was evaluated by ANOVA (analysis and variance). The model was developed using the software Design Expert v.13.0.12.0. The model in the form of ANOVA is shown in Table S4.

**Table S2.** Experimental range and levels of independient variables.

|  |  | **Range and levels** | | |
| --- | --- | --- | --- | --- |
| **Independent variable** | **Factor** | **-1** | **0** | **+1** |
| [Catalyst] (mg L^-1^) | A | 2 | 5 | 7 |
| [H_2_O_2_]_0_ (mg L^-1^) | B | 15 | 30 | 50 |

**Table S3.** CCRD design matrix and experimental results.

| **Run** | **A: [Catalys] (mg L^-1^)** | **B: [H_2_O_2_]_0_ (mg L^-1^)** | **R: k (min^-1^)** |
| --- | --- | --- | --- |
| 1 | 7 | 15 | 0.523 |
| 2 | 5 | 15 | 0.323 |
| 3 | 2 | 30 | 0.036 |
| 4 | 5 | 50 | 0.474 |
| 5 | 2 | 15 | 0.044 |
| 6 | 5 | 30 | 0.516 |
| 7 | 7 | 30 | 0.718 |
| 8 | 7 | 50 | 0.944 |
| 9 | 2 | 50 | 0.035 |


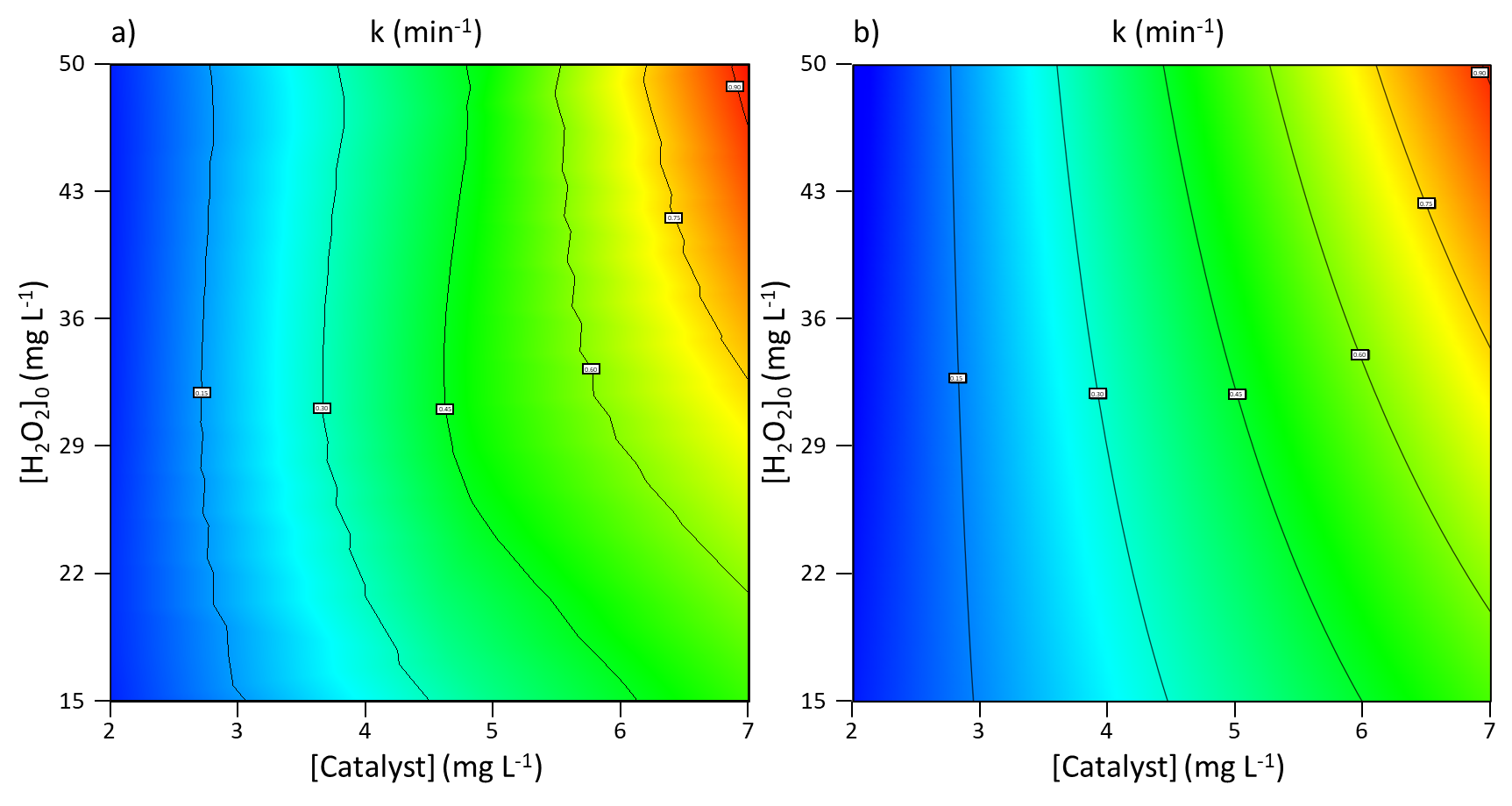


**Fig. S4.** Response surface of the apparent pseudo-first order kinetic constant in function of H_2_O_2_ and Fe(III) concentration at a Fe(III):EDDS molar ratio of 1:0.5, a) experimental results and b) two-factor interaction model.

**Table S4.** ANOVA test for response function.

| **Source** | **Sum of Squares** | **Degrees of Freedom** | **Mean Square** | **F-value** | **p-value** |
| --- | --- | --- | --- | --- | --- |
| Model | 0.357 | 3 | 0.119 | 91.66 | < 0.0001 |
| A | 0.326 | 1 | 0.326 | 251.19 | < 0.0001 |
| B | 0.017 | 1 | 0.017 | 13.03 | 0.0154 |
| AB | 0.019 | 1 | 0.019 | 14.47 | 0.0126 |
| Residual | 0.007 | 5 | 0.001 |  |  |
| Correlation Total | 0.363 | 8 |  |  |  |

The model F-value of 91.66 and a probability value (p-value) smaller than 0.0001 implied that it is significant. Also A, B and AB are significant model terms for CYN degradation. Moreover, the model allowed a succesfull prediction of the experimental values, with correlation coefficients around 0.96.

**Fig. S5.** Apparent pseudo-first order kinetic constant and H_2_O_2_ conversion values obtained in the photo-Fenton oxidation of CYN with LED on different aqueous matrix (operating conditions: [CYN]_0_ = 100 mg L^-1^; [H_2_O_2_] = 30 mg L^-1^; [Fe(III)] = 5 mg L^-1^; Fe(III):EDDS = 1:0.5 (molar ratio); pH_0_ ~ 7; T = 25 ºC).

**Fig. S6.** Evolution of CYN upon photo Fenton oxidation with different sources of irradiation ([CYN]_0_ = 100 µg L^-1^; [H_2_O_2_] = 30 mg L^-1^; [Fe(III)] = 5 mg L^-1^; Fe(III):EDDS = 1:0.5 (molar ratio); pH_0_ ~ 7; T = 25 ºC). Experimental (symbols) and model fit (solid lines).
